# Ultrafast Laser‐Driven Crystallization of LTA Zeolite

**DOI:** 10.1002/smtd.70929

**Published:** 2026-08-04

**Authors:** Meryem Merve Doğan, Mehdi Hagverdiyev, Sezin Galioglu

**Affiliations:** ^1^ UNAM—National Nanotechnology Research Center and Institute of Materials Science and Nanotechnology Bilkent University Ankara Türkiye; ^2^ UNAM—National Nanotechnology Research Center Bilkent University Ankara Türkiye

**Keywords:** CO_2_ adsorption, light‐material interactions, LTA framework, ultrafast laser synthesis, zeolites

## Abstract

In conventional synthesis routes, crystallization is governed by bulk heating, diffusion, and reactor‐scale heat/mass transfer. The primary challenge in these synthesis methods is not only achieving crystallization but controlling how the energy is delivered to the precursor medium, because the spatiotemporal distribution of energy largely determines nucleation and growth of crystals. Here, we provide an ultrafast laser synthesis method where a localized energy‐delivery is achieved for driving LTA zeolite crystallization directly from liquid precursor suspension. This method enables precise control over nucleation and growth. Hence, the resulting LTA zeolite crystals synthesized via the ultrafast laser method demonstrate uniform particle size distribution, 28% greater total surface area, 57% greater external surface area, and 30% enhanced CO_2_ adsorption (2.44 mmol g^−^^1^) compared to their hydrothermally synthesized counterpart, thanks to the dynamic environment of the synthesis method. Both XRD and FTIR characterizations show high crystallinity (90%) alongside the absence of detectable silanol or extra‐framework aluminum species, validating that the ultrafast laser method maintains complete structural integrity in the LTA framework despite rapid crystallization. This work establishes the ultrafast laser method as a powerful approach for producing high‐quality zeolite crystals with enhanced adsorption capacity, offering an alternative route to microporous materials in carbon dioxide capture and green chemical processes.

## Graphical abstract

The ultrafast laser synthesis technique, using femtosecond laser pulses, enhances zeolite synthesis by providing high‐precision control over energy delivery and reaction kinetics. This technique accelerates the synthesis of high‐quality zeolite crystals with enhanced functionality. This study investigates LTA zeolites with multiple aspects, including structural properties, crystal formation zone, defect generation, and CO_2_ adsorption behavior.

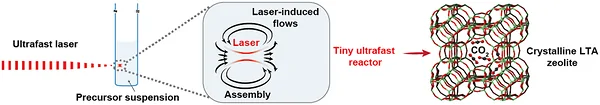

## 1 Introduction

Zeolites are crystalline aluminosilicates with uniform microporous pores and channels [1, 2, 3, 4]. They exhibit high specific surface area [1, 2, 3], tunable acid‐base centers [3, 5], and exchangeable cations [3, 6], leading to strong ion‐exchange capacity [6], and high thermal and chemical stability [7]. These properties make them widely used in petrochemical catalysis [8], detergents [9], gas sensors [10], biomedical applications [11], energy storage [12], and agriculture [13]. Their versatility in various applications has attracted growing attention toward the synthesis and precise control of diverse frameworks over the past decade. Conventional zeolite synthesis relies on the hydrothermal crystallization of an amorphous precursor suspension prepared and aged in a basic (pH > 10) medium. Zeolite crystals are synthesized from silica and alumina sources in a stainless steel/teflon‐lined autoclave under moderate temperatures (between 50°C and 270°C) and autogenous pressure [4, 14]. During the crystallization, thermal energy activates the reactions, initiating nucleation and growth, however, reaction time varies from several hours to days depending on framework type. Conventional heating limits precise energy control, which in turn restricts nucleation and growth control [15].

Unconventional synthesis methods such as microwave‐assisted, high energy UV and Gamma‐ray hydroxyl radical‐assisted, continuous flow and ultrasonic‐assisted synthesis aim to overcome these intrinsic shortcomings of the conventional hydrothermal method by changing the mode of energy delivery and/or heat‐ and mass‐transfer dynamics [16, 17, 18, 19, 20, 21, 22, 23, 24]. The limitations of these methods are associated with issues such as repeatability, process complexity, undesired mesopore formation, and safety concerns. In this regard, the ultrafast laser synthesis method, which we recently introduced, offers an innovative perspective to literature by simultaneously changing energy delivery and mass‐transfer dynamics aspects [25].

The ultrafast laser synthesis method, where multiphoton absorption generates laser‐induced flows, provides a dynamic synthesis environment. Laser‐induced flows ensure dynamic heat‐ and mass‐transfer, enabling accelerated nucleation and growth without undesired byproducts. Besides, ultrafast lasers ensure precise energy delivery to the reaction medium on the time scale of silica polymerization/depolymerization reactions (i.e., pico/femtoseconds) [26], distinguishing this approach from both conventional and unconventional methods like microwave‐assisted, or hydroxyl radical‐assisted synthesis [18, 19, 20, 21]. This controlled energy deposition allows the crystallization to freeze at that state by simply turning off the laser at any stage of the synthesis. Thus, intermediate stages can be studied to elucidate nucleation and growth mechanisms, as shown for the sequential evolution of TPA‐Silicalite‐1 synthesis from early oligomers to fully developed crystals in our previous study [25]. The spatially and temporally localized, precise energy delivery via multiphoton absorption makes the ultrafast laser method applicable to various zeolite frameworks.

Here, we extend our ultrafast laser synthesis approach to Na‐form aluminosilicate zeolite LTA (Na‐LTA), which exhibits a more complex crystallization mechanism than simple silicious TPA‐Silicalite‐1. This work investigates the LTA zeolites in depth from multiple aspects, including (i) structural properties, ***(ii)*** crystal formation zone ***(iii)*** defect generation, and ***(iv)*** CO_2_ adsorption behavior. LTA zeolites synthesized by hydrothermal synthesis (control samples) and ultrafast method were characterized by Scanning Electron Microscopy (SEM), X‐Ray Diffraction (XRD), High‐Resolution Transmission Electron Microscopy (HR‐TEM), Fourier Transformed Infrared (FTIR) Spectroscopy, Thermogravimetric Analysis—Differential Thermal Analysis (TGA‐DTA), and Brunauer‐Emmet‐Teller (BET) methods and CO_2_ capture analysis.

## 2 Results and Discussion

### 2.1 Synthesis Mechanism

Unlike continuous wave (CW) and nanosecond lasers, femtosecond laser pulses generate very steep local thermal gradients in the liquid (Figure 1A) [30]. Under femtosecond laser irradiation, once the optical breakdown threshold is exceeded (≥GW cm^−2^), typically initiated by multiphoton absorption [31], transient plasma, cavitation bubbles, shock waves, and high‐speed convective flows emerge [25, 31, 32]. The proposed ultrafast laser zeolite synthesis uses a unique energy delivery mechanism that localizes energy spatially and temporally to a very small volume (hot zone) at the glass‐liquid interface (Figure 1B and S1), eliminating the need for certain temperatures and pressure required in the conventional hydrothermal synthesis method [25].

**FIGURE 1:**
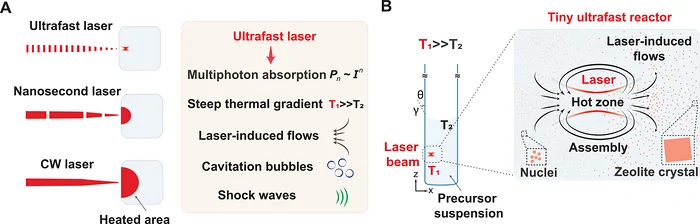
(A) Schematic illustration of the heat accumulation by different types of laser beam (left) and the events that occur during the ultrafast laser‐liquid interaction (right). *P*_n_ indicates n photon absorption probability, and *I* indicates laser intensity. (B) Schematic illustration of the ultrafast laser synthesis environment and tiny ultrafast reactor. T_1_ is the temperature in the hot zone, T_2_ is the temperature of the surrounding liquid (i.e., precursor suspension), *γ* is the surface tension, and *θ* is the angle of contact.

In this dynamic environment, silica polymerization‐depolymerization reactions, which form zeolite crystals [27, 28, 29], are initiated within the hot zone. The hot zone serves as a tiny ultrafast reactor where the laser pulses are absorbed through multiphoton absorption (Figure 1B). A transient plasma is formed as the multiphoton absorption triggers optical breakdown, resulting in cavitation bubbles [31, 33]. Local steep thermal gradients drive strong Marangoni flow that attracts nuclei and small clusters toward the hot zone. Building units and assembled clusters are constantly formed in the hot zone and dispersed throughout the precursor suspension via laser‐induced flows (Figure 1B). Both the formed cavitation bubbles and fast laser‐induced flows enhance the mixing of the precursor suspension. All these events contribute to ultrafast laser‐driven assembly and facilitate controlled nucleation and growth of zeolite crystals.

### 2.2 Properties of Synthesized Zeolite LTA

Characterizations of LTA zeolite nanocrystals synthesized via ultrafast laser and conventional hydrothermal methods were conducted to evaluate the impact of ultrafast laser synthesis methodology on the morphology, crystallinity, and thermal stability of the LTA zeolite nanocrystals.

The morphology of LTA zeolite nanocrystals synthesized via both methods was examined by SEM and it was observed that discrete, fully grown crystal structures were formed with cubic morphology expected for LTA zeolite (see Figures 2A and 2B) [34, 35, 36]. Although the average crystal size is < 300 nm (Figures 2A and 2B inset), a more well‐defined cubic morphology was observed for the ultrafast laser‐synthesized LTA crystals, similar to micron‐sized LTA crystals [36, 37]. This difference is attributed to variations in mass and heat transfer mechanisms in the dynamic synthesis environment of the ultrafast laser method compared to the static hydrothermal method.

**FIGURE 2:**
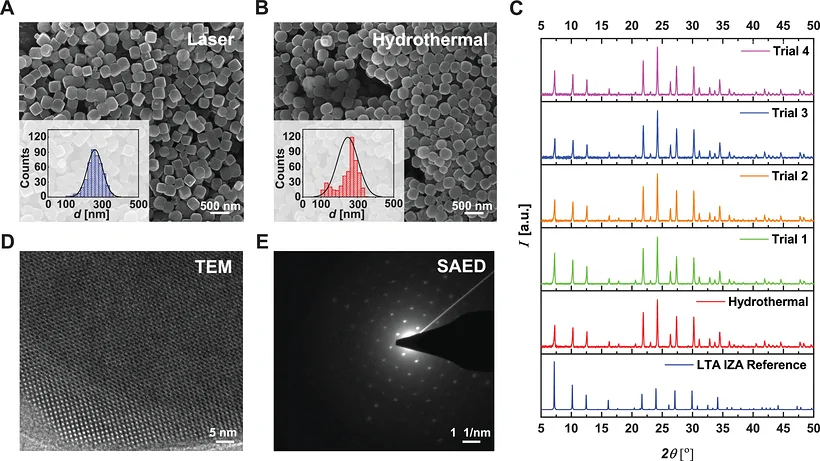
SEM images of Zeolite LTA crystals synthesized via (A) ultrafast laser method and inset shows the average crystal size distribution with *n* = 462, mean = 259.4 nm, standard deviation (SD) = 42.1 nm, and the coefficient of variation (CV = (SD/mean) × 100) = 16.3%, (B) conventional hydrothermal method and inset shows the average crystal size distribution with *n* = 462, mean = 247.3 nm, SD = 57.2 nm, and CV = 23.1%. (C) XRD patterns of ultrafast laser‐synthesized Zeolite LTA synthesized via the hydrothermal synthesis method and the ultrafast laser method, where the original experiment (Trial 1 with 100% relative crystallinity) was repeated after 1 week (Trial 2 with 97.5% relative crystallinity), 1 month (Trial 3 with 93% relative crystallinity) and 3 months (Trial 4 with 99% relative crystallinity). The patterns are compared to the reference XRD pattern approved by the International Zeolite Association (IZA). (D) HR‐TEM image and (E) Selected area electron diffraction (SAED) image of the single LTA zeolite crystal synthesized via ultrafast laser.

The average crystal sizes of LTA zeolite synthesized via the ultrafast laser method have a similar average crystal size (259.4 ± 42 nm, mean ± SD) with approximately 10 nm larger than those synthesized via the hydrothermal method (247.3 ± 57 nm, mean ± SD). The zeolite crystals synthesized via the ultrafast laser method exhibit a narrower distribution (CV = 16.3%, see Figure 2A inset) relative to their counterparts synthesized via the hydrothermal method (CV = 23.1%, see Figure 2B inset). It can be hypothesized that the nuclei and the assembled clusters are formed at similar time intervals. Mixing of liquid and balanced mass transfer due to fast laser‐induced flows continuously transferring the nuclei and the assembled clusters to the hot zone (see Figure 1B and Video S1). This ensures that growth is limited to existing crystals and uniform crystal sizes are obtained. Thus, the growth process proceeds in a controlled manner, and the resulting zeolite particles exhibit a narrower size distribution as observed in Figure 2A.

The relative crystallinity (%) of the LTA nanocrystals synthesized via both synthesis methods and the repeatability of the ultrafast laser method were examined by XRD analysis. XRD patterns in Figure 2C display that the crystal structure was successfully formed in each synthesis, with no detectable impurity phases. The relative crystallinity was determined as 100%, 97.5%, 93%, and 99% for Trials 1, 2, 3, and 4, respectively. Considering the four syntheses shown in Figure 2C and the repeated syntheses performed for other characterizations, it is evident that the ultrafast laser method is a reliable and reproducible synthesis method.

TEM analysis was also conducted to verify the structural nature of the synthesized zeolites. The lattice fringes and sharp SAED diffraction spots indicate crystalline domains, whereas ring‐like SAED features are characteristic of amorphous structures [38, 39]. The crystalline nature of the LTA zeolite synthesized via the ultrafast laser method was further confirmed by the lattice fringes observed in the HR‐TEM image in Figure 2D and diffraction spots observed in the SAED pattern in Figure 2E.

### 2.3 LTA Zeolite Crystal Formation Zone

Having established that femtosecond laser irradiation can directly crystallize LTA zeolite from a supersaturated precursor suspension, we next pursued to define the crystal formation zone and the operational window of laser parameters. Laser parameters such as pulse fluence, pulse width, wavelength, repetition rate, etc. were previously studied on different laser‐induced crystallization mechanisms, such as non‐photochemical laser‐induced nucleation (NPLIN), high‐intensity laser‐induced nucleation (HILIN), and laser trapping‐induced crystallization (LTIC) for simple organic crystals such as urea, glycine, L‐histidine, etc. [40, 41, 42]. Comprehensive characterization of these interdependent laser parameters is, therefore, essential to understand and control the physical (resulting size, morphology, and distribution, etc.) and chemical mechanism (nucleation rate, induction time, etc.) of laser‐driven crystallization of zeolite [25, 40]. It also facilitates the investigation of optimal processing conditions that enable reproducible synthesis with desired material properties.

To define the formation zone, eight systematic experimental trials (E_1_–E_8_) were conducted in which pulse fluence and repetition rate were systematically varied within the accessible laser parameter space while maintaining constant solution volume (400 µL) and approximately constant total energy input per mole of SiO_2_ (see Table S1 for details). We experimentally demonstrate that fully grown LTA zeolite crystals synthesized via the ultrafast laser method can be obtained only within a specific operating window defined by pulse fluence and repetition rate combinations, as in the formation zone diagram in Figure 3. Outside this region, crystallization is incomplete, resulting in milky or opaque white dispersions. Full precipitation of zeolite crystals occurs only after the crystals are fully grown (see Figure S2). This behavior highlights the necessity for sufficient average absorbed power density to surpass critical thresholds in both spatial and temporal domains, ensuring stable zeolite framework assembly.

**FIGURE 3:**
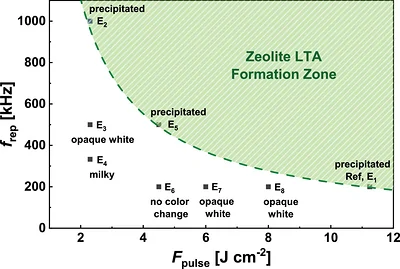
The formation zone map of ultrafast laser synthesis for LTA zeolite illustrates how pulse fluence (*F*_pulse_) and repetition rate (*f*_rep_) affect LTA zeolite nucleation and growth. The shaded region indicates the operating window in which fully LTA crystal formation (precipitation) is achieved. The dashed line is included for visual guidance.

The lower boundary of the formation zone (Figure 3 dashed curve) suggests that equivalent crystallization can be achieved through adjustments in laser pulse parameters. Under conditions of lower pulse fluence (2.30 J cm^−2^) and higher repetition rate (1 MHz) (i.e., E_2_ in Figure 3), the required average power density is achieved for complete crystallization. On the contrary, in a high pulse fluence‐low repetition rate case (i.e., E_1_ in Figure 3), lower repetition rate (200 kHz) but higher‐energy pulses (11.24 J cm^−2^) provide the necessary average absorbed power density. This consistency in crystallization behavior strengthens the idea that the effective control parameter for zeolite nucleation and growth is the average absorbed power density, which affects spatiotemporal energy delivery essential for polymerization reactions and framework assembly that forms zeolite crystals. Ultimately, crystallization kinetics are more responsive to average absorbed power (*P*_A_ in Table S1) than to integrated energy delivery (total energy delivery, *E*_tot_ in Table S1), allowing for the optimization of the operational window by targeting specific time‐averaged absorbed power densities while balancing pulse energy and repetition rate for desired synthesis results.

### 2.4 Defect Formation and Thermal Stability

Next, to investigate whether ultrafast laser processing induces any measurable framework defects, LTA zeolite crystals synthesized via both ultrafast laser and conventional hydrothermal methods are comparatively analyzed by FTIR spectroscopy. The low Si/Al ratio (Si/Al ≥ 1) of the aluminosilicate LTA mandates strict Si–O–Al alternation throughout the framework, as enforced by Löwenstein's rule prohibiting Al–O–Al linkages [14, 36, 43]. In Na‐form LTA zeolites, this negative framework charge is primarily balanced by extra‐framework Na^+^ cations. This structural ordering minimizes the accumulation of coordinatively unsaturated framework sites [44, 45, 46]. Therefore, the formation of silanol defects (≡Si–OH), commonly observed in high‐silica zeolites [44, 45], is generally not expected to be dominant or readily observable in Na‐LTA. Nevertheless, recent studies have shown that silanol defects can be introduced even in LTA zeolite when crystallization is carried out via alternative high‐energy irradiation‐based synthesis methods [21]. In FTIR spectra, framework defects of high silica zeolites are observed in the hydroxyl stretching region (3200–4000 cm^−1^), with sharp peaks particularly around 3740 and 3720 cm^−1^ for isolated external silanols and internal silanols, respectively [44, 45, 47].

As seen in the FTIR spectra in Figure 4A, the broad band at the hydroxyl stretching region (3200–4000 cm^−1^) is significantly diminished after the dehydration process. FTIR spectra of vacuum dried samples show no detectable bands corresponding to silanol defects (no sharp band in 3740 cm^−1^) in samples synthesized via both synthesis methods (Figure 4A), demonstrating that the rapid laser‐induced crystallization process does not compromise framework integrity. Similarly, in the FTIR spectra in Figure 4A, no distinguishable bands are observed in the ∼3770–3780 and ∼3660–3640 cm^−1^ regions, which are typically reported as Al‐containing extra‐framework hydroxyl species or partially framework‐bound/degraded Al sites [44, 45, 48, 49]. This provides no FTIR evidence for significant extra‐framework Al (Extra‐framework aluminum (EFAL) species) formation during crystallization or calcination at 500°C.

**FIGURE 4:**
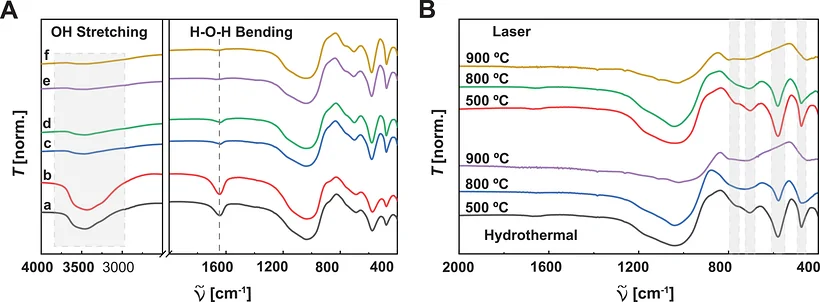
(**A)** FTIR spectra of calcined (at 500°C) LTA zeolite crystals **a**. synthesized via hydrothermal method and **b**. synthesized via ultrafast laser method measured at room temperature, **c**. synthesized via hydrothermal method and **d**. synthesized via ultrafast laser method measured after vacuum drying treatment at 150°C, **e**. synthesized via hydrothermal method, and **f**. synthesized via ultrafast laser method measured after vacuum drying treatment at 200°C. (B) FTIR spectra of LTA zeolite crystals, which were synthesized via ultrafast laser and hydrothermal methods, were calcined at 500°C, 800°C, and 900°C and were dehydrated overnight in a vacuum oven at 200°C after pellet preparation.

To evaluate the structural stability of zeolite LTA crystals synthesized via ultrafast laser and hydrothermal methods, FTIR analyses were performed on samples calcined at various temperatures (500°C, 800°C, and 900°C) (Figure 4B). The characteristic vibrations of the LTA zeolite framework include a strong, broad band of asymmetric stretching vibration of T–O bonds (Si–O–Si and Si–O–Al) in the 950–1050 cm^−1^ region, with symmetric stretching occurring around 650–750 cm^−1^ [47, 48, 49]. The double four‐membered ring (D4R) vibrations characteristic of the LTA framework topology are evident at approximately 550–560 cm^−1^, and the T–O bending vibrations appear at around 450–470 cm^−1^ [47, 49, 50]. These fundamental framework vibrations serve as fingerprints for the LTA zeolite structure.

The FTIR spectra in Figure 4B indicate gradual framework degradation at increased calcination temperatures, although the initiation and degree of degradation vary between synthesis methods. After calcination at 500°C, both ultrafast laser and hydrothermally synthesized samples preserve their distinctive framework vibrations observed by FTIR, as further confirmed by XRD analysis (see Figure S3). Thermogravimetric analysis (TGA‐DTA) results show similar thermal behavior for LTA zeolite samples synthesized via hydrothermal and ultrafast laser methods, with the sample mass stabilizing at 75.8% and 76.2%, respectively, after Tetramethylammonium (TMA) removal in the 400°C–500°C range (see Figure S4). After heat treatment at 800°C, FTIR spectrum of LTA crystals synthesized via hydrothermal method shows a significant band broadening and intensity attenuation in the D4R vibrations (575 cm^−1^), reduced band separation at 675–775 cm^−1^ region, and loss of resolution of the characteristic LTA features are observed, particularly in the 800–400 cm^−1^ region, while the same vibrations remain more clearly resolved in the LTA crystals synthesized via ultrafast laser method. Although, no evidence of phase transformation or secondary phase formation was observed in the XRD patterns of both samples treated at 800°C (see Figure S3), the hydrothermally synthesized LTA crystals showed a pronounced decrease in relative crystallinity (63.3% crystallinity) and in peak intensity (Figure 4B) in FTIR spectrum after treatment at 800°C compared to zeolite LTA synthesized via ultrafast laser method (82.4% crystallinity).

Upon increasing the temperature to 900°C, the characteristic framework vibration bands broaden significantly and lose their distinguishability in both samples (i.e., hydrothermal and laser samples). Furthermore, no diffraction peaks are observed in the XRD pattern in both samples (see Figure S3). These findings indicate framework distortion and structural collapse. The improved structural integrity of the LTA zeolite crystals synthesized via the ultrafast method provides enhanced thermal stability under thermal stress, which can be attributed to their defect‐free nature, within the detection limits of FTIR, and a higher degree of crystallinity. Although FTIR analysis did not reveal detectable framework defects, a definitive evaluation of the defect‐free nature of the framework would require complementary characterization techniques, such as solid‐state NMR spectroscopy.

### 2.5 CO_2_ Adsorption Behavior

Zeolites are widely studied for CO_2_ adsorption due to their large surface area, adjustable pore characteristics for different framework types, and strong affinity toward CO_2_ molecules, offering significant CO_2_ adsorption capacity at room temperature and low pressures (< 5 bar) [51, 52, 53]. Among them, LTA zeolite stands out due to its low Si/Al ratio. Presence of a high amount of Al^3^^+^ in the lattice creates a negative charge on each AlO_4_ tetrahedron [54], which is balanced by exchangeable extra‐framework cations (such as Na^+^ and K^+^) located within the pore system. CO_2_ adsorption in LTA zeolite occurs primarily through electrostatic interactions between these extra‐framework cations and the CO_2_ molecule [51, 55]. The small pore size (≃ 0.4 nm) and high cation density of the LTA structure allow the negatively charged oxygen atoms of CO_2_ to attract toward cations, thus localizing CO_2_ to specific cation sites within the pore [51, 55, 56]. Because the LTA structure contains three distinct extra‐framework cation sites (Sites I, II, and III as shown in Figure 5A), they allow CO_2_ to bind to multiple cations, particularly in a “bridging” geometry that enhances stability and adsorption capacity by coordinating two oxygen atoms of CO_2_ to separate cation sites [51].

**FIGURE 5:**
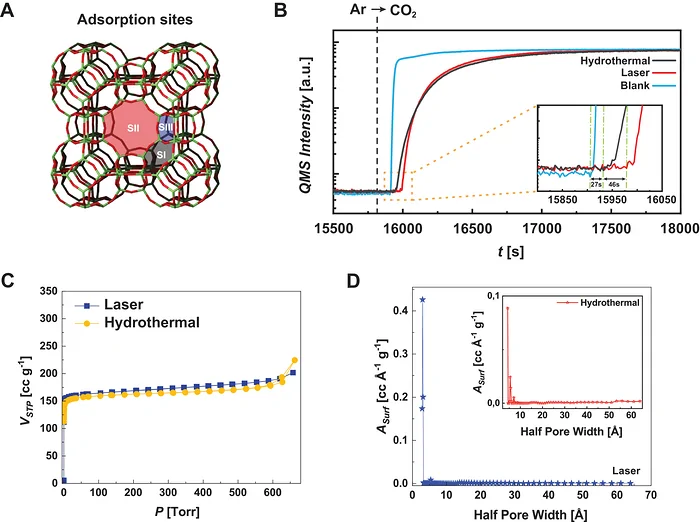
**(A)** Schematic illustration of the adsorption sites of the LTA zeolite framework. (B) Plot of dynamic breakthrough curves of blank test (blue), LTA zeolite synthesized via ultrafast laser method (red), and conventional hydrothermal method (gray). The right inlet is the zoomed plot of dynamic breakthrough curves. The green dashed lines are guidelines to see at what time the breakthrough point corresponds to. N_2_ adsorption‐desorption isotherms of uncalcined LTA zeolite crystals synthesized via (**C)** ultrafast laser method and conventional hydrothermal methods. (D) Plot of half‐pore width distribution for the LTA zeolite synthesized via ultrafast laser method, and its hydrothermally synthesized counterpart (inset).

When a new synthesis route is developed for zeolites, examining its impact on gas‐adsorption sites provides valuable insight into structure‐property relationships. Since variations in the energy deposition (e.g., ultrafast lasers vs. conventional hydrothermal heating) can significantly influence crystallization kinetics, morphology, accessible micro/mesoporosity, and surface area, these factors can directly affect CO_2_ binding strength and uptake [20, 21, 57, 58]. Therefore, we examined how the novel ultrafast laser synthesis conditions affect CO_2_ adsorption capacity of LTA zeolite crystals by using a dynamic breakthrough curve analysis in which CO_2_ signal was monitored over time by mass spectrometry (Figure 5B). The shape of the breakthrough curve provides important information about adsorption kinetics [51, 59, 60, 61]. In the analysis of breakthrough curves, three fundamental aspects are typically evaluated: (i) slope of the breakthrough curve, (ii) breakthrough delay, and (iii) saturation time.

Slope of the breakthrough curve indicates how rapidly adsorption occurs [51, 59]. The breakthrough curve of LTA zeolite synthesized via the ultrafast laser method exhibits a steeper slope, approximately 10% higher than the hydrothermal sample, suggesting faster mass transfer, thus faster adsorption kinetics. The breakthrough delay is the time from the moment CO_2_ gas is introduced into the system until the first CO_2_ signal is detected by the mass spectrometer [51, 52, 60]. During this time, CO_2_ molecules diffuse into the pores of the adsorbent material and bind to the active surface sites. Compared to the “blank sample”, the initial detection of CO_2_ by the mass spectrometer occurred at 27 s for the hydrothermal sample (Figure 5B inset) but was delayed by 73 s for LTA crystals synthesized via the ultrafast laser method. This difference in breakthrough delay indicates that the two synthesis methods produce LTA crystals with different pore accessibility and diffusion‐related mass transfer behavior. Saturation time is the time interval between the initial detection of the CO_2_ signal and the point where the outlet CO_2_ concentration aligns with the inlet concentration [61, 62, 63]. The saturation time of the hydrothermally synthesized sample is 40 min shorter than that of the sample synthesized via the ultrafast laser method, suggesting a lower capacity or weaker interaction with CO_2_. In accordance with these observations and findings, CO_2_ adsorption capacities are calculated as 2.44 mmol g^−1^ for LTA zeolite synthesized via ultrafast laser method and 1.87 mmol g^−1^ for hydrothermally synthesized LTA crystals (see SI for detail).

Additional N_2_ adsorption‐desorption analyses were conducted to further elucidate these pronounced differences in CO_2_ adsorption capacity since specific surface area, micropore volume and external surface area are the critical parameters because they directly affect surface related properties of zeolites, such as adsorption capacity and catalytic activity [64, 65]. The BET isotherm obtained for LTA zeolite in Figure 5C exhibits Type I isotherm behavior characteristic of microporous materials according to the IUPAC classification [69]. The N_2_ adsorption‐desorption results in Table 1 indicate that the BET specific surface area of ultrafast‐laser‐synthesized, and hydrothermally synthesized LTA zeolites are 675.01 and 648.39 m^2^ g^−1^, respectively. These values are consistent with the specific surface area ranges reported in the literature for LTA zeolites with different Si/Al ratios and confirm that the samples have a highly porous structure [64, 66, 67, 68]. The, DFT‐based surface area of of LTA zeolites synthesized via ultrafast laser and hydrothermal methods are 931.6 and 725.7 m^2^ g^−1^, respectively. According to the *t*‐plot method, the LTA crystals synthesized via both methods exhibited the same micropore volume (0.23 cm^3^ g^−^^1^), whereas DFT analysis revealed micropore volumes of 0.28 and 0.27 cm^3^ g^−^^1^ for the ultrafast laser and hydrothermal methods, respectively. In the literature, the enhanced CO_2_ adsorption capacity is attributed to the combined contributions of extra‐framework cation‐CO_2_ interactions [51, 55], increased micropore volume [61, 70], and higher total and external surface areas [61, 70, 71]. As summarized in Table 1, LTA crystals synthesized via both methods have same Si/Al ratio. However, LTA crystals synthesized via ultrafast laser method have 28% higher DFT‐based total surface area, 3.7% higher micropore volume and 57% higher external surface area. To further investigate the origin of the increased external surface area, the total pore volume was determined from the N_2_ adsorption‐desorption isotherm. The total pore volume was found to be 0.31 cm^3^ g^−^^1^, whereas the micropore volume was 0.28 cm^3^ g^−^^1^. This small difference, together with the Type I adsorption isotherm, the absence of an apparent mesopore peak in the pore size distribution (Figure 5D), and the lack of a hysteresis loop at high relative pressures, suggests that significant mesopore formation did not occur. Therefore, the observed increase in external surface area is more likely associated with morphological differences, such as reduced crystal intergrowth and well‐defined crystal edges observed in the SEM images, and a more uniform particle size distribution confirmed by the particle size distribution analysis, rather than mesopore formation. DFT‐based pore size distribution of ultrafast‐laser‐synthesized LTA zeolites in Figure 5D reveals a pore size uniformity with a half‐pore width of 2.998 Å, comparable to molecular‐scale dimensions characteristic of the zeolite LTA framework.

**TABLE 1:** Summary of synthesis parameter, structural and textural properties, and CO_2_ adsorption performance of LTA zeolite crystals synthesized via ultrafast laser and conventional hydrothermal method.

Method	*t*_rxn_ [h]	Si/Al Ratio^a^	Crys.^b^ [%]	*d_crystal_* ^c^ [nm]	*S_DFT_* ^d^ [m^2^ g^−1^]	*V_micro_* ^e^ [cm^3^ g^−1^]	*S_ext_* [m^2^ g^−1^]	*q_CO2_* ^f)^ [mmol g^−1^]	*τ_delay_* ^g^ [s]
L	4	1.6	99.4	∼ 259	931.6	0.28	60.13	2.44	73
H	8	1.6	100	∼ 247	725.7	0.27	38.38	1.86	27

^a^ The Si/Al ratio obtained from the average of seven SEM‐EDX analysis. The obtained Si/Al ratio is compatible with synthesis conditions containing organic structure directing agent TMA.

^b^ The relative crystallinity is calculated based on the five most intense characteristic peaks from the XRD pattern of LTA zeolite compared to its hydrothermally synthesized counterpart..

^c^ The average crystal size determined by the analysis of 462 individual LTA crystals from SEM images using ImageJ software..

^d^ The surface area was determined by DFT analysis.

^e^ The micropore volume was determined by DFT analysis.

^f^ CO_2_ adsorption capacity is calculated from the area under the dynamic breakthrough curve..

^g^ Breakthrough delay determined as the time difference between the first detectable increase in QMS signal in the blank and zeolite measurements.

Abbreviations: S_ext_, External surface area; H, Hydrothermal; L, Laser.

The steeper dynamic breakthrough profile and the higher adsorption capacity clearly demonstrate that the LTA zeolites synthesized via the ultrafast laser method possess enhanced textural (Figure 5 and Table 1) and structural properties, resulting in more effective interactions with CO_2_.

## 3 Conclusion

In conclusion, LTA crystals synthesized via the ultrafast laser method exhibit a consistently superior combination of structural, morphological, and functional properties compared with their hydrothermally synthesized counterparts. LTA crystals synthesized via ultrafast laser method shows a defect‐free framework within the sensitivity of the FTIR method with enhanced thermal stability and a high degree of crystallinity (>90%), suggesting that the ultrafast laser‐driven crystallization pathway promotes the formation of a highly ordered LTA framework with better structural resilience under thermal treatment. The steeper dynamic breakthrough profile and the higher adsorption capacity clearly demonstrate that the LTA zeolites synthesized via the ultrafast laser method possess enhanced textural and structural properties, resulting in more effective interactions with CO_2_. Under sufficiently high absorbed power density, spatiotemporally localized energy deposition, fast laser‐induced flows and dynamic condition of the synthesis results in crystals with a uniform size‐distribution, a lower degree of intergrowth, and a higher surface area. LTA crystals synthesized via ultrafast laser method have 28% higher DFT‐based total surface area, 3.7% higher micropore volume and 57% higher external surface area. The small difference between total pore volume (0.31 cm^3^ g^−^^1^) and micropore volume (0.28 cm^3^ g^−^^1^), together with the Type I adsorption isotherm, the absence of an apparent mesopore peak in the pore size distribution, and the lack of a hysteresis loop at high relative pressures, suggests that significant mesopore formation did not occur. Therefore, the observed increase in external surface area is more likely associated with morphological differences, such as reduced crystal intergrowth and well‐defined crystal edges observed in the SEM images, and a more uniform particle size distribution confirmed by the particle size distribution analysis, rather than mesopore formation. The structural and functional improvements indicate that the ultrafast laser method enhances the performance of well‐studied zeolites and offers significant engineering potential for other structures. Accordingly, future studies on ultrafast laser method can focus on scalability of the synthesis for practical applications, integrating advanced spectroscopic monitoring for real‐time analysis, and extending the methodology to diverse zeolite frameworks.

## 4 Methods

### 4.1 Ultrafast Laser Setup

LTA zeolite syntheses were carried out in a vial (Isolab, N13 vial, 4 mL) with inserts (Restek, WISP‐48, 500 µL) that functioned as a closed system integrated with a femtosecond laser (Spectra Physics, Spirit One, 1040–8 SHG). The laser's central wavelength was 1040 nm, and the pulse repetition rates were set to 200 kHz, 500 kHz, and 1 MHz. The laser beam was directed into the reaction vial, which was suspended in air by attaching a sample holder to its cap (Figure S1). The beam follows the optical path by impinging on a mirror that directs it to two consecutive lenses. Then, it is directed to a half‐wave plate and a beam splitter that facilitates beam attenuation for power regulation. Finally, the beam passes through a 1‐inch lens with a focal length of 5 cm and reaches the reaction vial, where the beam is focused on the glass‐liquid interface. The beam diameter was measured to be approximately 9 µm using the Thorlabs beam profiler.

### 4.2 Chemicals Used

Tetraethylorthosilicate (TEOS, 98%, Sigma Aldrich), aluminum isopropoxide (Al(iPro)_3_, >98%, Sigma Aldrich), tetramethylammonium hydroxide (TMAOH, 25 wt. %, Sigma Aldrich), deionized water (DI H_2_O, resistivity = 18.2 MΩ), and sodium hydroxide (NaOH, 99%, Merck) were used as received.

### 4.3 Synthesis of LTA Zeolite

LTA zeolites were synthesized from the precursor suspension with molar composition: *Al*(*iPro*)_3_ : 3 *TEOS* : 7.36 *TMAOH* : 0.33 *NaOH* : 192.41 *H*_2_ *O*. To prepare precursor suspension, DI water (0.95 g) was added to TMAOH (2.17 g, 25 wt%) and stirred for 20 min. Subsequently, Al(iPro)_3_ (0.17 g) was slowly added to the mixture and stirred for 1 h. TEOS (0.53 g) was then added, and the mixture was stirred for 2 h. In a separate beaker, NaOH (0.01 g) and DI water (0.23 g) were mixed and added dropwise to the final mixture. Finally, the mixture was aged at room temperature for 15 h under stirring. After aging, 400 µL of the precursor suspension was placed in an oven at 100°C for 8 h for hydrothermal treatment. Another 400 µL of the precursor suspension was ultrafast laser‐treated (Spectra‐Physics, 200 kHz repetition rate, 5 W average laser power unless stated otherwise) for 4 h. After ultrafast laser treatment, the samples were separated into precipitated and supernatant parts. Precipitated parts were washed four times with DI water to lower the pH to ∼7 by centrifugation (14000 rpm, 10 min, Eppendorf Centrifuge 5420), and dried overnight at 45°C.

### 4.4 Characterization

*X‐ray diffraction patterns of the powder LTA zeolite* were measured by Malvern Panalytical X'Pert Pro Multipurpose X‐ray diffractometer with Cu Kα radiation (operating at 45 kV, 40 mA) in the 2θ range of 5°–50°. The percent crystallinity was estimated using the sum of the relative intensities of the 5 most intense peaks of the LTA zeolite synthesized via ultrafast laser method and the hydrothermal method.

*The morphology* of the LTA crystals was analyzed by scanning electron microscopy (SEM, FEI Quanta 200F, 30 kV accelerating voltage, 10 nm Au/Pd coated). Si/Al ratios of the Na‐LTA crystals were determined by Energy‐dispersive X‐ray spectroscopy (Genesis Apex 4 EDAX coupled with Quanta FEI 200F SEM, at 10 kV voltage with a spot size of 4) at least from 7 different spots. The average crystal size was determined from SEM images by taking an average of at least 300–400 zeolite particles per sample using ImageJ software.

*SAED pattern* was obtained by High‐resolution Transmission Electron Microscopy (FEI TECNAI G2 20 S TWIN TEM, at high tension of 200 kV). To prepare the sample for TEM analysis, zeolite powder was suspended in ethanol, sonicated for 5 min, drop‐cast onto a copper grid, and dried under ambient conditions.

*Thermogravimetric‐differential thermal analysis (TG‐DTA)* for thermal stability analysis, zeolitic water, and structure directing agent (TMA) contents determination was performed using SDT650 TGA‐DSC (at 25°C–900°C temperature range, heating rate of 5°C/min). Differential thermal analysis was obtained by taking the differential of the TGA graphs using OriginLab Software.

*The Brunauer‐Emmett‐Teller (BET) method* was applied to determine the total surface area of the calcined LTA crystals (at 500°C for 3 h with the heating rate of 5°C/min) was determined by utilizing the Quantachrome Autosorb IQ2 MP Gas Sorption system (employing N_2_ adsorption‐desorption isotherm at 77.3 K). Before measurement, the sample underwent outgassing at 300°C for 16 h. The specific surface area was calculated using the BET method and DFT analysis and the micropore volume were obtained by *t*‐plot analysis and DFT analysis. The external surface areas were obtained by *t*‐plot analysis. The pore size distribution was obtained by density functional theory (DFT) analysis, as DFT provides a more detailed description of pore widths in microporous materials compared to conventional methods based on capillary condensation. The amount of product obtained in a single ultrafast laser synthesis experiment is approximately 7 mg. Therefore, multiple equivalent batches prepared under the same synthesis conditions were characterized by XRD and subsequently combined. The N_2_ adsorption‐desorption data was obtained from a combined sample prepared from multiple independent synthesis batches, and statistical uncertainty estimates could not be determined due to the lack of independently replicated adsorption measurements. The total pore volume analysis was conducted on the adsorption isotherm data using the Quantachrome instrument software by assigning the V tag to the last adsorption data point in the isotherm.

*Fourier Transform Infrared (FTIR) spectroscopy* was performed to investigate the functional groups and vibrational characteristics of the samples. Measurements were carried out in transmission mode using a Bruker TENSOR 37 spectrometer operated via OPUS software. For sample preparation, LTA zeolite powder was finely ground and mixed with spectroscopic‐grade potassium bromide (KBr, >99%, Sigma‐Aldrich) at a 1:100 weight ratio. The mixture was ground in an agate mortar and pestle and pressed into transparent pellets using the hydraulic press (Carver, 4350L) under approximately 8 tons pressure for 3–5 min. The pellets were analyzed in the mid‐infrared region from 4000 to 400 cm^−1^ with a spectral resolution of 4 cm^−1^. Samples were calcined at 500°C, 800°C, and 900°C for 3 h and dehydrated overnight in a vacuum oven at 200°C after pellet preparation. Following drying, the samples were cooled from 200°C to 80°C in the vacuum oven and immediately transported into a small desiccator to the measurement setup, where measurements were conducted at room temperature under ambient conditions.

*CO_2_ capture measurements* were conducted using the CatLab‐2, a fully automated catalyst characterization system by Specson Instruments. It is equipped with five Bronkhorst Mass Flow Controllers and a quartz fixed‐bed tubular reactor, connected to an SRS RGA 200 mass spectrometer for real‐time gas analysis. The system also features an integrated furnace capable of reaching temperatures up to 900°C for high‐temperature pre‐treatments. To capture CO_2_, calcined (at 500°C for 3 h with the heating rate of 5°C/min) 100 mg of each sample (LTA zeolite) synthesized via ultrafast laser and conventional hydrothermal method is placed inside a quartz reactor, and each sample undergoes thermal activation before adsorption tests. This involves heating the sample to 350°C with an argon flow for 2 h, cooling to ambient temperature, and stabilizing under continuous argon flow for 60 min. A mixture containing 2% CO_2_ in argon gas is introduced into the reactor at a flow rate of 20 sccm, maintaining room temperature and pressure for 3 h. The mass spectrometer continuously tracks the *m/z* 44 signal, providing real‐time data for dynamic breakthrough curves. The amount of product obtained in a single ultrafast laser synthesis experiment is approximately 7 mg. Therefore, multiple equivalent batches prepared under the same synthesis conditions were characterized by XRD and subsequently combined. The CO_2_ adsorption data was obtained from a combined sample prepared from multiple independent synthesis batches, and statistical uncertainty estimates could not be determined due to the lack of independently replicated adsorption measurements.

## Author Contributions

Sezin Galioglu designed the research and supervised the study. Meryem Merve Doğan performed the experiments and characterizations. Mehdi Hagverdiyev performed characterizations. Sezin Galioglu and Meryem Merve Doğan analyzed the data. Sezin Galioglu and Meryem Merve Doğan wrote the paper. All authors read and discussed the final manuscript.

## Funding

TUBITAK ARDEB 3501 project with number 123F473.

## Conflicts of Interest

The authors have declare no conflicts of interest.

## Supporting information




**Supporting File 1**: smtd70929‐sup‐0001‐SuppMat.docx.

**Supporting File 2**: smtd70929‐sup‐0002‐VideoS1.mp4.

## Data Availability

The data that support the findings of this study are available from the corresponding author upon reasonable request.
