# Supplementary material for: Ultrafast Laser‐Driven Crystallization of LTA Zeolite
Source: Small Methods. 2026 Aug 4;10(21):e70929. doi: 10.1002/smtd.70929 (PMC13745981; doi:10.1002/smtd.70929)
Supplement: Supplementary file 1 [file SMTD-10-e70929-s001.docx]

Supporting Information

Ultrafast Laser-Driven Crystallization of LTA Zeolite

Meryem Merve Doğan, Mehdi Hagverdiyev, Sezin Galioglu*

M. M. Doğan, M. Hagverdiyev

UNAM - National Nanotechnology Research Center and Institute of Materials Science and Nanotechnology, Bilkent University, Ankara, 06800, Türkiye

E-mail: [meryem.dogan@bilkent.edu.tr](mailto:meryem.dogan@bilkent.edu.tr), [m.hagverdiyev@bilkent.edu.tr](mailto:m.hagverdiyev@bilkent.edu.tr)

S. Galioglu

UNAM - National Nanotechnology Research Center, Bilkent University, 06800, Ankara, Türkiye

E-mail: [sezin@unam.bilkent.edu.tr](mailto:sezin@unam.bilkent.edu.tr).

1. Experimental Set-up Used for Ultrafast Laser Synthesis Method

**Figure S1.** Laboratory photograph and a schematic of the optical setup used for ultrafast laser synthesis method.

2. LTA Zeolite Crystal Formation Zone Experiments

The "milky" regime in Figure 3 and Figure S2 corresponds to the early nucleation stage, where the suspension exhibits slight turbidity, indicating early stage of crystal formation where extended time required for complete crystallization. The "opaque white" regime represents a synthesis stage where crystal growth is dominant. The "precipitated" state implies complete crystallization, wherein LTA crystals have settled as a powder phase at the bottom, leaving a transparent supernatant above. This progressive transition from blurry to milky to precipitated states reflects the evolution of the crystallization process and provides visual indicator that the synthesis is complete.

**Figure S2**. Representative images of reaction vials from different stages of LTA zeolites’ crystal formation. In the experiments, a 45 x14.75 mm (IsoLab, N13 vial) was used as the outer container and a 40 x 8.2 mm (Synthesis Lab, La Pha Pack Vial 1 mL) insert was used as the inner container.

The absorbed laser power was determined by analyzing transmission losses through the glass vial–glass insert–precursor suspension system, while accounting for reflection, absorption, and scattering at the relevant optical interfaces. The transmittance of the vial and insert walls was first measured under unfocused conditions, followed by focused measurements to evaluate losses originating from the glass insert and the glass insert–liquid interface. From these measurements, the average input power reaching the precursor suspension and the corrected output power emerging from it were calculated, and their difference was taken as the absorbed power. This analysis showed that approximately 26.4% of the incident average laser power was absorbed by precursor suspension with minor fluctuations arising from plasma formation and fluid dynamics during the measurements. Using this absorbed fraction, the absorbed pulse energy (*E*_abs_) and absorbed pulse fluence (*F*_pulse_) were calculated for each condition in Table S1. The zeolite crystallization is primarily influenced by the average absorbed power density, rather than individual pulse characteristics.

**Table S1.** Table presents the effect of laser parameters on the crystallinity and yield of laser-synthesized zeolite LTA crystals.

| **No** | ***V***  **[µl]** | ***P*_I_ [W]** | ***P*_A_  [W]** | ***F*_p_  [J cm^-2^]** | ***E*_abs_  [µJ]** | ***t***  **[h]** | ***f* [kHz]** | ***E*_Tot_**  **[MJ per** $\boldsymbol{mol}_{\boldsymbol{SiO}_{\boldsymbol{2}}}$**]** | | ***C***  **[%]** | ***Y***  **[mg]** |
| --- | --- | --- | --- | --- | --- | --- | --- | --- | --- | --- | --- |
| **E1** | **400** | **5** | **1.43** | **11.24** | **7.15** | **4h** | **200** | | **84.12** | **100** | **6.8** |
| **E2** | 400 | 5.4 | 1.46 | 2.30 | 1.46 | 3h 55 min | 1000 | | 84.12 | 96.7 | 5.4 |
| **E3** | 400 | 3.5 | 0.73 | 2.30 | 1.46 | 7h 50 min | 500 | | 84.12 | 91.9 | 6.3 |
| **E4** | 400 | 2 | 0.49 | 2.30 | 1.46 | 11h 41 min | 333 | | 84.12 | 95.2 | 2.3 |
| **E5** | 400 | 5 | 1.43 | 4.50 | 2.86 | 4h 1 min | 500 | | 84.12 | 93.1 | 3.0 |
| **E6** | 400 | 2.5 | 0.57 | 4.50 | 2.86 | 10h 2 min | 200 | | 84.12 | - | - |
| **E7** | 400 | 3.6 | 0.76 | 6 | 3.82 | 7h 32 min | 200 | | 84.12 | 95.0 | 3.6 |
| **E8** | 400 | 4.2 | 1.018 | 8 | 5.09 | 5h 38 min | 200 | | 84.12 | 94.8 | 4.4 |

*V* - Volume, *P*_I_ - incident average laser power, *P*_A_ - absorbed average laser power, *F*_p_ - pulse fluence, *E*_abs_ – absorbed pulse energy, *t* - time, *f* - frequency, *E*_Tot_ - total deposited energy, *C* - crystallinity, *Y* - yield, *T* - trial. The relative % crystallinity was calculated based on the reference E1 sample.

3. XRD Patterns of LTA Crystals After Heat Treatment

**Figure S3**. XRD patterns of LTA crystals synthesized via **(a)** ultrafast laser method and **(b)** conventional hydrothermal synthesis method after calcination at different temperatures.

4. Thermogravimetric Analysis (TGA) – Differential Thermal Analysis (DTA)

**Figure S4**. Plots of thermogravimetric analysis (TGA) and differential thermal analysis (DTA) of LTA crystals synthesized via **A)** ultrafast laser method and **B)** conventional hydrothermal synthesis method.

5. CO_2_ Adsorption Measurements.

Prior to measurements, a thermal activation treatment was carried out to eliminate water molecules from the pores, guaranteeing that CO_2_ molecules could fully enter the pore space. As observed from thermogravimetic analysis (TGA) in **Figure S4**, the water retention of LTA crystals synthesized via the ultrafast laser method and the hydrothermal method was quite similar. Following the thermal activation, the samples were exposed to a mixture of 2% CO_2_ in argon gas at 1 bar and 20 sccm flow rate at room temperature for 3 hours. During this time, dynamic breakthrough curves were obtained by continuously monitoring the m/z 44 signals (i.e., CO_2_ signal) with a mass spectrometer. The lack of a signal detection for a certain period despite the introduction of CO_2_ gas into the system indicates that CO_2_ is diffusing into the pores of the adsorbent and is being retained. During the period when this signal is not observed, CO_2_ is adsorbed by the adsorbent in the active sites; when the signal begins to increase, the system reaches saturation. Therefore, the area under the breakthrough curve provides quantitative information about the adsorption capacity.

To quantitatively calculate the CO_2_ adsorption capacity, the system was first calibrated. For this purpose, a "blank test" was conducted under the same experimental conditions with an empty reactor containing no adsorbent, and system induced lag times were determined. This process is critical to isolate the effect of the adsorbent alone. Firstly, the calibration area (S), which is the theoretical signal area obtained by introducing CO_2_ at a constant flow rate under known flow conditions, is determined:

$$\begin{aligned} S=\frac{P.V_{total}}{R.T} \#\left( 1 \right) \end{aligned}$$

where P is the atmospheric pressure, V_total_ is the total calibration volume, R is the ideal gas constant, and T is the temperature. The calibration volume is the amount of CO_2_ introduced into the system without adsorbent during the experiment:

$$\begin{aligned} V_{\mathrm{total}}= Time (min) \times Flow rate (\mathrm{cm}^{3}/min) \times\frac{\% CO_{2}}{100} \#\left( 2 \right) \end{aligned}$$

When the parameter values used in the experiment are included, the calibrated volume is calculated as:

$$\begin{aligned} V_{\mathrm{total}}=10 min \times20 \frac{cm^{3}}{\min} \times\frac{2}{100}=4 cm^{3}\#\left( 3 \right) \end{aligned}$$

When we put this volume value into Equation 1, the calibrated area value is:

$$\begin{aligned} S=\frac{1\times4\times{10}^{-3}}{0.082057 \times298.15} \simeq1.635 \times{10}^{-4}\mathrm{mol}=0.1635\mathrm{mmol}\#\left( 4 \right) \end{aligned}$$

Secondly, the CCA, the area under the CO_2_ breakthrough curve obtained by the mass spectrometer in the experiment, is calculated. This area was calculated for each measurement using OriginLab Software. The actual adsorption is calculated as the ratio of this area to the calibration area (S) and normalized by the mass of the adsorbent:

$$\begin{aligned} Captured CO_{2}=\frac{CCA}{S} \times\frac{1g}{m} \#\left( 5 \right) \end{aligned}$$

where m is the mass of the adsorbent and it is 0.1 g in our work. In the calculations, the sample weight was accepted as less than 0.1 g based on TGA analysis due to the amount of water removed from the pores (see Figure S4).

When CCA is found by subtracting the area under the breakthrough curve of the laser or hydrothermal sample from the area under the breakthrough curve of the "blank test", the CO_2_ adsorption capacity for the laser sample is calculated as 2.44 mmol g^-1^ and the CO_2_ adsorption capacity for the hydrothermal sample as 1.87 mmol g^-1^.
